# Self-Efficacy, Proxy Efficacy, Media Literacy, and Official Media Use in COVID-19 Pandemic in China: A Moderated Mediation Model

**DOI:** 10.3389/fpsyg.2022.847522

**Published:** 2022-05-12

**Authors:** Qingrui Li, Yu Zheng, Junqing Zhang, Rui Geng

**Affiliations:** Faculty of Humanities and Arts, Macau University of Science and Technology, Taipa, Macao SAR, China

**Keywords:** media literacy, proxy efficacy, mediation effect, moderation effect, self-efficacy

## Abstract

**Purpose:**

COVID-19 pandemic is a significant threat toward the public health. However, the discussion of the mechanism of media literacy’s effect in fighting against pandemic is limited. Thus, this study aims to explore the mechanism with a sociocognitive perspective.

**Methods:**

A survey was administrated to 420 college students in China. PROCESS macro of SPSS was applied to analyze the data and test the moderated mediation effect.

**Results:**

The moderated mediation model of media literacy, proxy efficacy, self-efficacy, and official media use was tested and supported. Official media use was a negative moderator on the association between media literacy and proxy efficacy.

**Conclusion:**

The study explored the media literacy’s role as a determinant of proxy efficacy and self-efficacy, which contributed to the sociocognitive theory.

## Introduction

The impact of the COVID-19 pandemic has gone beyond the original prediction of people in both size and time aspects. Cooperation between the public and governments is required. The public’s confidence in themselves and governments is necessary to achieve this. In spreading pandemic-related information, both useful and scientific one and misinformation are of large amounts. To distinguish them, media literacy is required (Howell and Brossard, 2021).

Given that people went out less during the pandemic, media became essential information sources (Liu et al., 2021). In China, official media is the authority of pandemic-related information publishing with higher credibility of information. Therefore, the coverage of official medial could play an essential role in public information gaining and confidence improving.

The aim of the study is to explore the determinants of self-efficacy of fighting against the COVID-19 pandemic under the sociocognitive theory. Based on the works of previous researchers, the current study collected data by questionnaires, then established a theoretical model to discuss the association between the public’s media literacy, proxy efficacy, self-efficacy, and official media use. The proxy efficacy’s mediation effect between media literacy and self-efficacy and the moderation effect of official media use between media literacy and proxy efficacy was verified. Also, the suggestion of improving the confidence of fighting against the pandemic.

## Literature Review

### Theoretical Framework: Sociocognitive Theory

#### Media Literacy and Self-Efficacy

Based on previous works, media literacy is defined as individuals’ ability to obtain, interpret, critically think about information, and improve daily life and social development (Yu and Zhao, 2017).

According to Bandura (2000), self-efficacy refers to individuals’ perceived ability to achieve certain goals. Individuals acquire cognitions about the society and environment by constant learning and practice, then articulate experience, which is an essential source of self-efficacy (Usher and Pajares, 2008). Media literacy could enhance an individual’s ability to obtain knowledge and then favor articulating cognition and experience (Austin et al., 2012).

Cakmak (2013) found that individuals’ self-efficacy of computer use improved after media literacy intervention. Furthermore, the association between media literacy and the self-efficacy of the public has been verified in health studies (e.g., Cho et al., 2020). In the public health area, self-efficacy is seen as the key role in improving people’s wellbeing (Cattell, 2001). Another study showed that for subjects correctly self-diagnosed with H1N1, their media literacy could positively predict self-efficacy (Avery, 2009). With the improvement of media literacy, the self-efficacy of the public for dealing with the pandemic can also be improved. That is, media literacy could be a determinant of self-efficacy.

With the outbreak of the COVID-19 pandemic, an infodemic is also emerged (Mheidly and Fares, 2020). Living with information of different qualities, media literacy of the public can represent their ability to obtain and understand information, then evaluate its credibility, which is a crucial source of self-efficacy (Avery, 2009). Therefore, in COVID-19 pandemic, media literacy can help people in their confidence of fighting against the pandemic by obtaining scientific protections.

The positive association between media literacy and self-efficacy has thus emerged in the studies mentioned above. In the COVID-19 pandemic, media literacy could help deal with the infodemic to obtain more credible information. Then, the confidence in coping with the pandemic could be improved. Therefore, we propose hypothesis 1.

*H1*: Media literacy can positively predict self-efficacy in protecting herself or others from the pandemic.

#### Proxy Efficacy as the Mediator

Social cognition theory argues that behaviors are determined by the interaction between social, environmental, and individual factors (Bandura, 1988). The individual factor’s role is based on the individual’s ability to handle events actively and strong expectancy for particular outcomes (Usher and Pajares, 2009). According to the social cognitive theory, individuals are no longer viewed as passive subjects whose behavior is reinforced by external stimuli alone but as active ones who have agency over their learning and the consequences of their actions (Bandura, 2001). In this regard, individuals’ level of trust in their ability to accomplish their goals (i.e., self-efficacy) and their levels of trust in the ability of third parties to assist them in accomplishing their goals (i.e., proxy efficacy) are both important sources of their efficacy (Bandura, 2000).

##### Media Literacy and Proxy Efficacy

Proxy efficacy refers to an individual’s perception of the ability of a third party (i.e., proxy) to help her accomplish a specific goal (Bandura, 2001), and higher proxy efficacy implies a higher level of trust in the proxy. Proxy efficacy in this study refers to the level of trust individuals have in the government’s ability to help them through the epidemic.

The vital role of media literacy in promoting citizens’ political participation and enhancing their right to information has gradually become a consensus among scholars (Craft et al., 2017; Tully and Vraga, 2018). Also, enhancing the public’s media literacy will help strengthen their interest and understanding in joining the discussion of political issues, thus increasing individuals’ level of trust in the government’s ability to do so (Burroughs, 2009; Kahne et al., 2012). In public health emergencies, increased proxy efficacy helps the public adopt scientific responses; that is, the role of media literacy in enhancing agent efficacy can help the public adopt more scientific strategies when encountering the epidemic (Smith and Rimal, 2009; Li, 2018). Cho et al. have conducted a remote media literacy intervene, which found that participants whose media literacy was enhanced by the intervention had significantly improved proxy efficacy, which reduced their unhealthy tanning behaviors (Cho et al., 2020).

The current study argues that in the context of the COVID-19 pandemic, the increased media literacy of the audience can help them learn more about the government’s effective prevention and control measures in the epidemic, thus increasing their sense of agent efficacy regarding the government’s ability to prevent the epidemic, leading to research hypothesis 2.

*H2*: Media literacy can positively predict an individual’s proxy efficacy in Chinese government.

##### Proxy Efficacy and Self-Efficacy

Efficacy is defined as an individual’s perceived ability to control aspects of her life (Bandura, 1986). Self-efficacy emphasizes one’s perception of herself, while proxy efficacy emphasizes the perceived ability to control a certain proxy (e.g., teachers, churches, and government).

It should be noticed that the relationship between self-efficacy and proxy efficacy is not exclusive but an interactive one. In different situations, different efficacies show different levels’ influence (Bandura, 2001). In situations with sufficient social resources and infrastructure, self-efficacy usually plays a stronger role than poxy efficacy; while self-efficacy and proxy efficacy function jointly when resources, especially public health resources, is insufficient (Smith and Rimal, 2009).

The existing literature has demonstrated that proxy efficacy positively predicts self-efficacy (Bray and Cowan, 2004; Bray et al., 2006), that is, the higher the proxy efficacy, the higher the self-efficacy (Bandura, 1997). In the context of bodybuilding, the proxy efficacy of fitness individuals (fitness instructors as the proxy in this study) significantly and positively predicted their self-efficacy, which in turn increased their participation in fitness classes (Bray et al., 2001). Furthermore, the public’s proxy efficacy regarding politics was also significantly and positively related to their self-efficacy for political information seeking (Farman et al., 2018).

The collective perspective provides an idea to explain the operation of proxy efficacy, which helps individuals perceive practical help from third parties in developing their self-efficacy, thus contributing to their self-efficacy (Kim et al., 2021). From the beginning of the pandemic to the present-day normalization of prevention and control, audiences are often confronted with many reports with different qualities from the media. Then, audiences with higher trust in the pandemic prevention policies of the basic level government, that is, higher proxy efficacy, are more likely to perceive the sense of order and stability brought by the proxy, thus enhancing their self-efficacy in the face of the pandemic. In other words, the higher the proxy efficacy, the higher the self-efficacy of individuals in the pandemic prevention and control. Thus, we propose hypothesis 3.

*H3*: Proxy efficacy can positively predict an individual’s self-efficacy in protecting herself or others from the pandemic.

##### The Mediation Effect of Proxy Efficacy

Studies above identified two pathways through which media literacy affects self-efficacy. More importantly, media literacy can also enhance the public’s self-efficacy by increasing proxy efficacy. In the context of public health emergencies, proxy efficacy strengthens the public’s confidence in their response to crises by enhancing their perceptions of organizational and institutional competence, which helps them adopt scientific responses (Li, 2018). Previous research has identified the mediation effect of proxy efficacy, where the level of the public’s social capital, through their sense of proxy efficacy for community leaders, indirectly influences their HIV prevention behaviors (Smith and Rimal, 2009). Thus, the current study argues that media literacy indirectly enhances individuals’ self-efficacy in responding to the pandemic by increasing their proxy efficacy, leading to research question 1.

RQ1: Is there a mediation effect of proxy efficacy between media literacy and self-efficacy?

### The Moderation Effect of Official Media Use

Media Mobilization Theory (MMT), as a positive effects view (Norris, 1999), emphasizes the positive effect of media use on public participation in political perceptions, arguing that media can enhance the public’s level of political perceptions and thus its perceived trust in government (Shah et al., 1999). There is a positive correlation between media use and public perceptions of agent efficacy regarding government (Avery, 2009), and there is a virtuous cycle between media use, public perceived trust in government, and political participation (Norris, 2000).

Official media is a concept proposed for China’s media context in the context of the new media environment (Huang et al., 2017; Sun, 2021) and refers to media majorly sponsored by the Party and government (Ma, 2021) or mass media under official control (Xue et al., 2018). Furthermore, official media use refers to the audience’s behavior of using official media. The image of the government or government officials shaped by official media affects the public’s perception of the government’s image and trust in government agencies (Huang et al., 2017). As official media use increases, the public’s level of trust in the government then rises, thereby increasing its sense of proxy efficacy in terms of government competence. In the case of the COVID-19 pandemic, Chinese official media tended to report on the COVID-19 pandemic using positive frames such as “effective control of the epidemic,” while negative frames that exaggerated the negative effects were often avoided (Zhang and Fleming, 2005: p. 327). Such positive frames constructed by official media helped increase Internet users’ level of political trust in the government (Xue et al., 2018).

The audience’s use of official media tends to make them share a consistent ideological outlook (Zhou and Lu, 2008), making China’s official media powerfully effective in political mobilization and popular education, which has positive implications for maintaining high levels of public trust in the government (Zhu, 2001). The current study argues that for audiences with low media literacy, if they have a high level of official media use, raising the media literacy of such audiences with the “help” of official media use will more significantly increase their level of confidence in the government’s ability to fight the pandemic. For individuals with higher media literacy, their proxy efficacy is higher, and official media use can help them further maintain their high level of proxy efficacy. As a result, we propose research question 2.

RQ2: Is there a moderating effect of official media use between media literacy and proxy efficacy?

**Figure 1:**
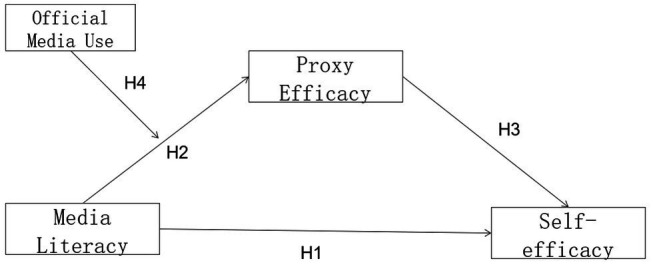
The theoretical model.

Also, based on media mobilization theory and the powerful effects of official media use, this study further infers that when official media use increases to a certain level, audiences are more likely to enhance proxy efficacy due to official media use and less likely to rely on media literacy. That is, official media use negatively moderates the relationship between media literacy and agent efficacy, leading to hypothesis 4 and the theoretical model shown in Figure 1.

*H4*: Official media use negatively moderates the relationship between media literacy and proxy efficacy.

## Materials and Methods

### Sampling

This study used a survey method, using the Wenjuanxing platform^1^ to form a questionnaire and through the WeChat Moments, snowball sampling to obtain convenient samples in mainland China and Macao S.A.R., and finally obtained 420 samples. The privacy and voluntariness of each participant was ensured in the data collection process. Due to the sample size of this study, the Boots-trapping method was used to draw samples during data analysis repeatedly, and the number of samples drawn was set at 5,000 to assess better the validity of the model constructed by the study.

### Measures

#### Media Literacy

Based on the previous research (Austin et al., 2016), this study measured respondents’ agreement with 10 statements such as “I would consider the intentions of the publisher of the article,” “I would compare information from different sources for the same event,” and “I would search for more relevant information to determine whether the content of an article is credible” (1 = strongly disagree, 6 = strongly agree) to measure respondents’ level of media literacy (*M* = 4.648, *SD* = 0.8506, *α* = 0.908).

#### Self-Efficacy

This used existing scales (Austin et al., 2012, 2021) and uses questions including “I can protect myself from coronavirus,” “I know how to protect myself from coronavirus,” “I know what to do if I have symptoms of coronavirus,” and “I know how not to protect others if I have coronavirus” (1 = very unlikely, 6 = very likely) to measure the self-efficacy of the respondents in the face of coronavirus (*M* = 4.7851, *SD* = 0.8709, *α* = 0.839).

#### Proxy Efficacy

Based on the literature (Liu et al., 2021), this study measured respondents’ proxy efficacy (*M* = 4.7667) through four questions: “I believe the community is confident in controlling the COVID-19 pandemic,” “I believe the community is capable of helping people through the COVID-19 pandemic,” “I believe the community is I believe that the community is well prepared to respond to the COVID-19 pandemic,” and “I believe that the community is skilled in using various methods to respond to the COVID-19 pandemic” were used to measure respondents’ proxy efficacy (*M* = 4.7667, *SD* = 1.0132, *α* = 0.957).

Official media use was measured by 2 items with 6-point Likert scale: (1) The frequency of using official media (1 = never, 6 = very frequent); and (2) I believe information offered by official media (1 = strongly disagree, 6 = strongly agree). Answers were multiplied to construct the variable (Ma and Wang, 2015). Higher scores of the variable indicate higher levels of official media use.

#### Control Variables

This study included demographic variables including gender, grade, and current usual residence in the model as control variables. Among them, males were coded as 1 and females were coded as 0; grades were coded as 1–8, representing freshman to doctoral students, respectively; the current usual residence was partially coded as 1 and 0, representing usual residence in mainland China and usual residence in Hong Kong, Macao, Taiwan, and overseas regions, respectively.

### Data Analysis

SPSS and its PROCESS macro were used to analyze data and test moderating and mediating effects (Hayes, 2017). The test answered this study’s two research questions, and all four research hypotheses were verified. Table 1 showed the results of descriptive statistics for the four main variables in this study.

**Table 1:** Descriptive statistics of major variables.

	High	Low	***M***	***SD***
Media literacy	6	1	4.648	0.8506
Self-efficacy	6	1	4.7851	0.8709
Proxy efficacy	6	1	4.7667	1.0132
Official media use	36	1	22.9381	8.5039

## Results

### Sample Characteristics and Descriptive Statistics of Key Variables

The total sample size is 420. Among them, most were female (71.43%) and 28.57% were male; 62.85% were undergraduates and 37.15% were graduates.

Table 2 showed results of the media literacy questionnaire among college students, where it was found that the college students were more concerned about the accuracy of media content (*M* = 4.81, 4.91; *SD* = 1.084, 0.985) and the timeliness of articles (*M* = 4.83, *SD* = 1.069), which indicated that the respondents have the higher ability in judging the timeliness and content of information. However, respondents paid less attention to the production process (*M* = 4.22, *SD* = 1.304), a gate-keeping mechanism (*M* = 4.52, *SD* = 1.219), and information source diversity (*M* = 4.45, *SD* = 1.234), and the degree of concern varied more. It meant that respondents’ literacy in judging information production is relatively low. That is, when exposed to media information, respondents showed higher media literacy regarding the content of the information itself. However, they were not very alert when thinking about and judging the information production and distribution parties and information production processes.

**Table 2:** Media literacy of respondents.

Question	***M***	***SD***
I would consider how this article was produced when reading content related to the COVID-19 pandemic (same below).	4.22	1.304
I would think about the source of this article.	4.72	1.206
I would consider the intent of the article publisher.	4.52	1.219
For the same event, I would compare information from different sources.	4.70	1.167
I would pay attention to the completeness and clarity of the original information.	4.81	1.084
When new developments occur, I decide whether to trust the new information by comparing it to previous information.	4.76	1.078
I would search for more relevant information to determine if the content of an article is credible.	4.45	1.234
I think it is important to think over and over about the message of the article.	4.56	1.116
I will consider whether the information in the article is accurate.	4.91	0.985
I will check the timeliness of the article.	4.83	1.069

### The Mediation Effect of Proxy Efficacy Between Media Literacy and Self-Efficacy

In this study, proxy efficacy was used to mediate media literacy and self-efficacy. Tables 3, 4 demonstrated the relationship between the variables in the mediating effect. Table 3 presented the direct effect of media literacy on self-efficacy and the indirect effect of media literacy on self-efficacy through proxy efficacy. Table 4 presented the specific indexes of the mediating effect’s total, direct, and indirect effects.

**Table 3:** The mediation effect of proxy efficacy between media literacy and self-efficacy.

Media literacy	Proxy efficacy		Self-efficacy	
	*β*	Boot SE	*β*	Boot SE
Constant variables	2.4664^***^	0.4186	2.6261^***^	0.2212
Media literacy	0.5002^***^	0.0738	0.3164^***^	0.0426
Proxy efficacy			0.4607^***^	0.0410
Sex	0.0067	0.1024	−0.0809	0.0632
Grade	−0.0416^*^	0.0233	−0.0078	0.0157
Residence	0.1401	0.1733	0.0152	0.0904
*R* square	0.1884		0.5201	
*F*	24.0851^***^		89.7469^***^	

^*^p < 0.05, ^***^p < 0.001.

**Table 4:** Direct effects, indirect effects and overall effect.

	Effect	Boot SE	Boot LLCI	Boot ULCI
Overall effect	0.5468	0.0432	0.4351	0.6585
Direct effect	0.3164	0.0390	0.2154	0.4173
Mediation effect: low official media use	0.2339	0.0408	0.1185	0.3376
Mediation effect: medium official media use	0.1760	0.0348	0.0954	0.2752
Mediation effect: high official media use	0.1181	0.0395	0.0325	0.2344

Data analysis showed that: (1) media literacy significantly and positively predicted proxy efficacy (*β* = 0.3820, *p* < 0.001); (2) media literacy significantly and positively predicted self-efficacy (*β* = 0.3164, *p* < 0.001); and (3) proxy efficacy significantly and positively predicted self-efficacy (*β* = 0.4607, *p* < 0.001); media literacy indirectly and positively predicted self-efficacy through proxy efficacy (*β* = 0.1953). Therefore, hypotheses 1, 2, and 3 were verified. Table 4 showed the total, direct, and indirect effects of the mediated model, and the analysis showed that effects above are within the 95% confidence interval, and the upper and lower bounds of the effects do not include 0, indicating that the model is a mediation model that answers research question 1.

The result suggested that the higher the media literacy of college students, the stronger their self-efficacy in coping with the pandemic when faced with information related to the COVID-19 pandemic. Also, media literacy indirectly affected self-efficacy through proxy efficacy. When media literacy increased, the more the group trusts the ability of the state and government to control the pandemic, thus increasing their confidence in their ability to cope with the pandemic.

### The Moderation Effect of Official Media Use Between Media Literacy and Proxy Efficacy

Table 5 showed the changes in the mediation model after the introduction of official media use and the moderation effect of official media use between media literacy and proxy efficacy. It was found that official media use significantly and positively predicted proxy efficacy (*β* = 0.0279, *p* < 0.001) and then negatively moderated the association between media literacy and agent efficacy (*β* = −0.0148, *p* < 0.01); the mediation model index after adding the moderating variable was −0.0068 [SE = 0.0023, 95% CI = (−0.0105, −0.0014). 0.0014]. Thus, research hypothesis 4 was validated and answered research question 2: there is a statistically significant moderation effect of official media use between media literacy and proxy efficacy.

**Table 5:** The moderation effect of official media use between media literacy and proxy efficacy.

IV	Proxy efficacy	
	*β*	Boot SE
Constant variables	4.8069^***^	0.1976
Media literacy	0.3820^***^	0.0670
Official media use	0.0279^***^	0.0059
Int: media literacy^*^ official media use	−0.0148^**^	0.0049
Sex	0.0214	0.0972
Grade	−0.0451^*^	0.0227
Residence	0.1647	0.1580
*R* square	0.2565	
*F*	23.7492^***^	

^*^p < 0.05, ^**^p < 0.01, ^***^p < 0.001.

**Figure 2:**
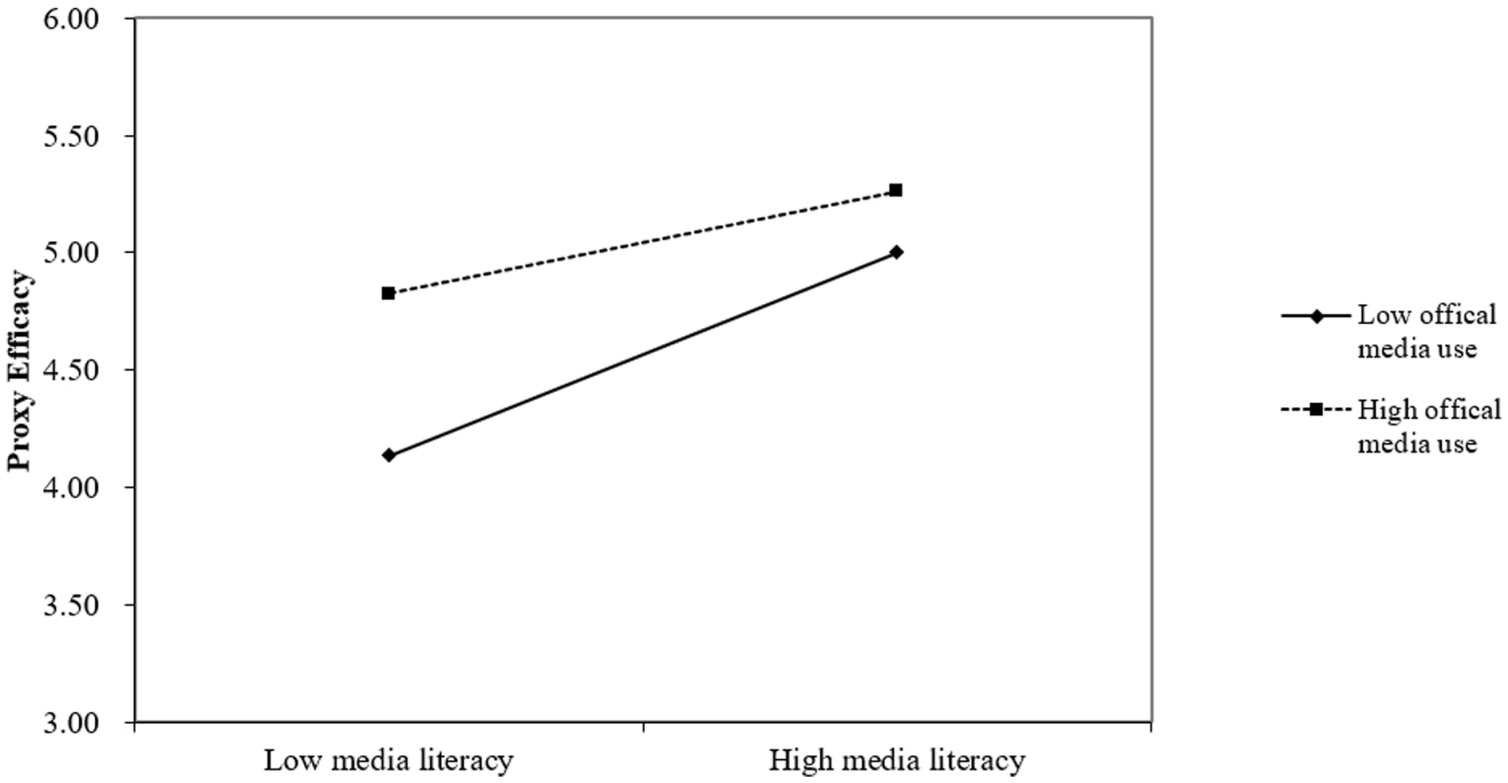
The moderation effect.

To further explore the moderation effect of the use of official media, Figure 2 demonstrated the changes in the influence between media literacy and proxy efficacy moderated by different levels of official media use. As can be seen from the figure, the slope increased from the high (M + 1SD) to low (M – 1SD) official media use group. When the degree of official media use is high, the positive relationship between media literacy and proxy efficacy is significant (*β* = 0.2563, *t* = 3.7196, *p* < 0.01). The positive relationship between media literacy and proxy efficacy was significant when official media use was low, and the effect of media literacy on proxy efficacy was more significant (*β* = 0.5077, *t* = 7.7402, *p* < 0.001).

**Figure 3:**
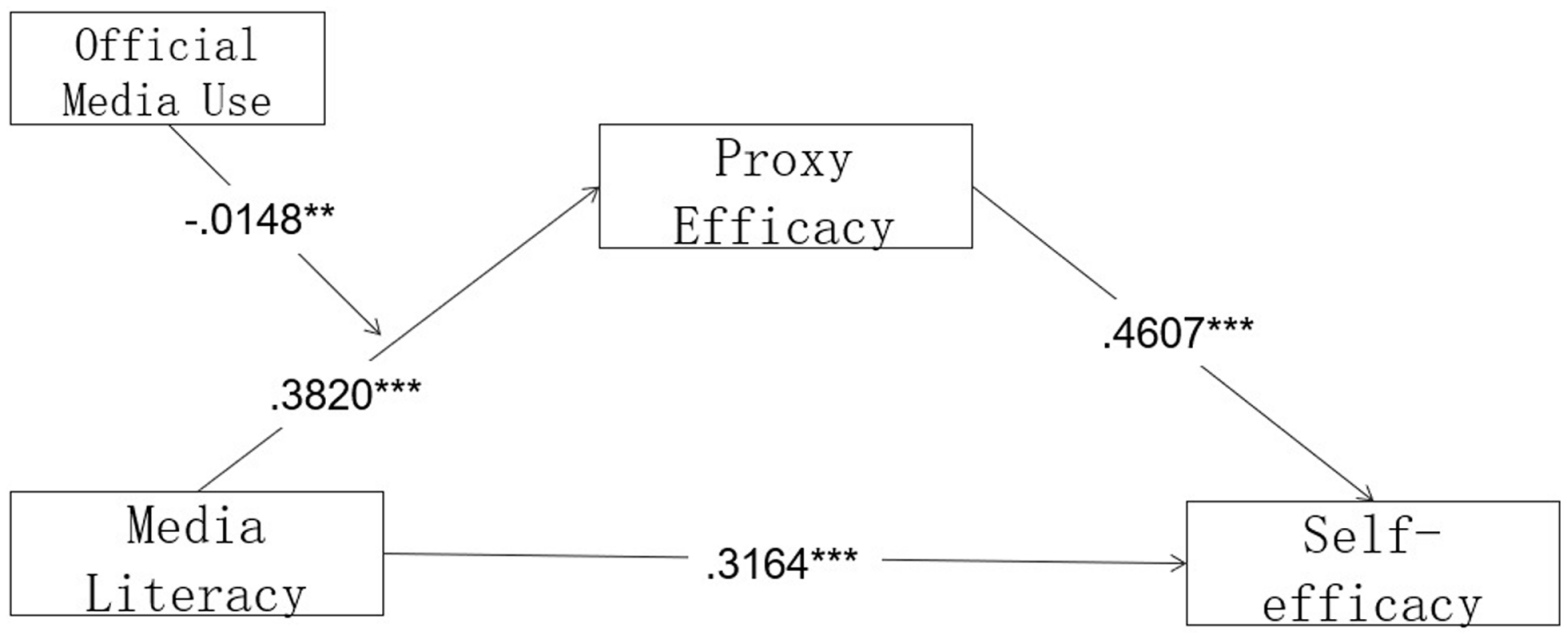
Model with coefficients.

This suggested that for respondents who use official media more, their proxy efficacy increased with increased use of official media, while at the same time, the effect of media literacy on their increased proxy efficacy is relatively weaker [SE = 0.0689, 95% CI = (0.1208, 0.3987)]. Whereas for the respondents who use official media less, the increase in media literacy was associated with a more significant increase in proxy efficacy [SE = 0.0656, 95% CI = (0.3788, 0.6367)]. Figure 3 shows the tested theoretical model and standardized regression coefficients.

## Discussion

### Theoretical Implications

The current study used a reliable measure of media literacy and enriched the empirical studies related to media literacy. In addition, this study also contributed in different aspects.

First, this study confirmed the critical role of public media literacy in responding to public health emergencies. Major findings of the study were in comply with previous works, which is, media literacy was positively associated with self-efficacy in health aspects (e.g., Avery, 2009; Cakmak, 2013). The theoretical model proposed in the present study confirmed that improving public media literacy will help the public take scientific strategies for pandemic prevention and control. The higher the media literacy of an individual, the greater her self-efficacy to protect herself and others in the pandemic. Also, the increased self-efficacy will help the public adopt scientific health maintenance behaviors (Yeun et al., 2013; Roncoroni et al., 2019; Choi, 2020; Bektas et al., 2021). This study provides empirical support for previous findings through empirical analysis and found a mediation effect of proxy efficacy between media literacy and self-efficacy, thus enriching previous theoretical models.

Another contribution of the current study was the discovery of the mediation effect of proxy efficacy. The results of this study demonstrated that an increase in individual media literacy could directly lead to an increase in self-efficacy. At the same time, media literacy can also indirectly affect self-efficacy through proxy efficacy. It has been suggested that in some specific situations, self-efficacy cannot be formed without the help of proxy (Kim et al., 2021): when individuals believe that what they are facing is beyond their ability to handle or are unwilling to face it alone, they choose to trust the ability of a third party, that is, proxy, and hope to use its power to achieve the desired goal (Bandura, 2001). In China, the “state” and “government” are considered responsible for controlling the pandemic, and the “state” and “government” are seen as a proxy to seek help. In China, where the “state” and “government” are considered responsible for controlling the epidemic, the “state” and “government” are seen as proxies, and help-seeking becomes common in the China context (Liu et al., 2021). Therefore, trust in the state and government’s ability to control the pandemic contributes to individuals’ confidence in preventing and controlling the pandemic. This study extended the works above in the Chinese context.

Notably, in public health emergencies, increased proxy efficacy helps the public adopt scientific strategies (Smith and Rimal, 2009; Li, 2018). That is, even without considering self-efficacy, the enhancement effect of media literacy on proxy efficacy can also serve to enhance the public’s level of scientific strategies when faced with an outbreak. To identify determinants of self-efficacy was an extension of studies mentioned above.

Finally, this study emphasized the importance of official media use in enhancing public efficacy. In China’s media system, political logic is the main rule the media industry follows. Traditional official media have also become a digital tool for the Party and government to enhance their governance capacity in the Internet era of media convergence (Xie and Song, 2021). The theoretical model proposed in the current study illustrated that official media use negatively moderated the relationship between media literacy and proxy efficacy. That is, groups with high levels of official media use have greater trust in government competence, while high or low levels of media literacy do not significantly affect their levels of proxy efficacy. To our knowledge, this study is the first to test the actual effect of official media on efficacy.

### Practical Implications

This study provided a new path to increasing individuals’ trust with different levels of media literacy in the government and other proxies. For the more media-literate group, whose efficacy is already high, increasing their use of official media can further increase their confidence in the government to help them through difficult times. However, the existing literature suggests that individual differences in the media literacy levels of new citizens in urban China differentiate them (Song, 2019) and that there is more room for improving the media literacy of college students in general (Chen, 2020; Yuan, 2020). The current study found that for individuals with lower levels of media literacy, increasing their official media use can more significantly enhance their trust in the government’s competence.

In other words, although improving public media literacy and developing media literacy education is important and long-term work, increasing the coverage of official media, promoting its image, and improving the public’s use of official media can be a complementary way to strengthen the public’s proxy efficacy. Thus, enhancing the confidence of some public with relatively low media literacy in themselves and the country can be achieved.

This study also has some limitations. First, the sample size is relatively small because the sample is a convenience sample, and the sample size of the female is relatively high; future studies can further improve the sample representativeness on this basis. Second, this study did not distinguish between types of media when measuring official media use; future studies can further refine the media types on this basis. Finally, the theoretical model of this study did not involve measurement of the behavior; future studies can research behavior and develop the theoretical model proposed in this study.

## Data Availability Statement

The raw data supporting the conclusions of this article will be made available by the authors, without undue reservation.

## Author Contributions

QL contributed to the conception, data analysis, and manuscript writing of the study. YZ contributed to parts of the conception, data analysis, and manuscript writing of the study. JZ contributed to the literature obtaining and analysis, manuscript writing, and performed the analysis with constructive discussions of the study. RG contributed to the literature obtaining and analysis, manuscript writing, and performed the analysis with constructive discussions of the study. All authors contributed to the article and approved the submitted version.

## Conflict of Interest

The authors declare that the research was conducted in the absence of any commercial or financial relationships that could be construed as a potential conflict of interest.

## Publisher’s Note

All claims expressed in this article are solely those of the authors and do not necessarily represent those of their affiliated organizations, or those of the publisher, the editors and the reviewers. Any product that may be evaluated in this article, or claim that may be made by its manufacturer, is not guaranteed or endorsed by the publisher.

## Footnotes

^1^ www.wjx.com
